# Supplementary material for: Identification of photoperiod-induced specific miRNAs in the adrenal glands of Sunite sheep (Ovis aries)
Source: Front Vet Sci. 2022 Jul 22;9:888207. doi: 10.3389/fvets.2022.888207 (PMC9354845; doi:10.3389/fvets.2022.888207)
Supplement: Supplementary file 1 [file Data_Sheet_1.ZIP › Supplementary file/miRDeep2 file.zip!/miRDeep2 file/Know_Structure/SP42A3/oar-mir-154b.pdf]

miRBase precursor : oar-mir-154b
Total read count : 874
oar-miR-154b-5p read count : 801
oar-miR-154b-3p read count : 73
remaining reads : 0

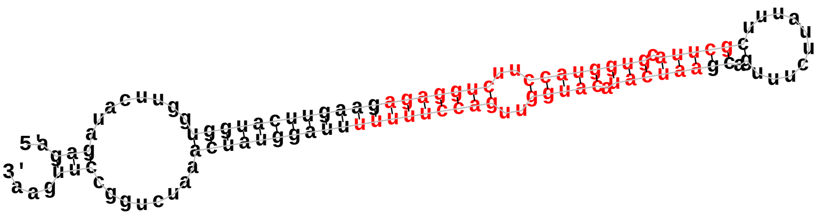

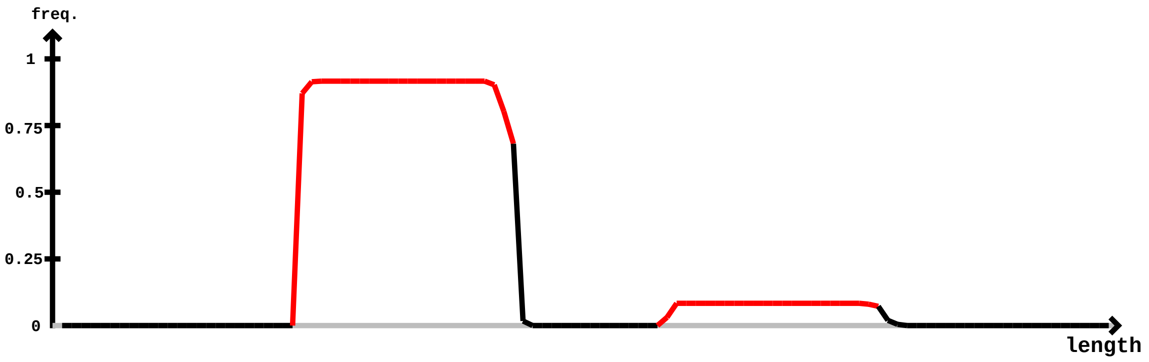


```
                                                                      oar-miR-154b-3p
                         oar-miR-154b-5p
5'- agagauacuuggugguacuugaagagaggucuuccauggugcauucgcuuuauucuuugacgaaucauacaugguugaccuuuuuuuagguaucaaaucuggccuugaa   -3'    exp
    .(((........(((((((((((((((((((..((((((((.((((((..........).)))))))).)))))..)))))))))))))))))))........)))...    reads       mm          sample
    ........................agaggucuuccauggugcau.................................................................    12          0           seq
    ........................agaggucuuccauggugcauu................................................................    85          0           seq
    ........................agaggucuuccauggugcauuc...............................................................    102         0           seq
    ........................agaggucuuccauggugcauucg..............................................................    547         0           seq
    ........................agaggucuuccauggugcauucgc.............................................................    15          0           seq
    .........................gaggucuuccauggugcauu................................................................    2           0           seq
    .........................gaggucuuccauggugcauuc...............................................................    4           0           seq
    .........................gaggucuuccauggugcauucg..............................................................    32          0           seq
    ..........................aggucuuccauggugcauucg..............................................................    2           0           seq
    ..............................................................aaucauacaugguugaccuuu..........................    3           0           seq
    ..............................................................aaucauacaugguugaccuuuu.........................    6           0           seq
    ..............................................................aaucauacaugguugaccuuuuu........................    14          0           seq
    ..............................................................aaucauacaugguugaccuuuuuu.......................    4           0           seq
    ...............................................................aucauacaugguugaccuuu..........................    1           0           seq
    ...............................................................aucauacaugguugaccuuuuu........................    32          0           seq
    ...............................................................aucauacaugguugaccuuuuuu.......................    9           0           seq
    ...............................................................aucauacaugguugaccuuuuuuu......................    4           0           seq
```
